# 全基因组芯片筛查非小细胞肺癌组织多药耐药相关基因

**DOI:** 10.3779/j.issn.1009-3419.2010.04.10

**Published:** 2010-04-20

**Authors:** 美燕 刘, 春红 李, 安 闫, 莉 蔡

**Affiliations:** 150001 哈尔滨，哈尔滨医科大学附属肿瘤医院 Department of Medical Oncology, the Affiliated Tumor Hospital, Harbin Medical University, Harbin 150040, China

**Keywords:** 肺肿瘤, MTT法, 全基因组芯片, 多药耐药, Lung neoplasms, MTT, cDNA microarry, Multi-drug resistant

## Abstract

**背景与目的:**

筛查与非小细胞肺癌多药耐药相关的基因，为非小细胞肺癌的个体化治疗提供理论依据。

**方法:**

将手术切除的肺癌组织细胞进行原代培养。首先，采用MTT法检测诺维本、吉西他滨、多西他赛、紫杉醇及顺铂对非小细胞肺癌组织细胞的抑制率和敏感性。再利用全基因组芯片筛选人高度敏感组和耐药组间的差异表达基因。

**结果:**

共筛选出差异表达基因212个，与耐药组相比，高度敏感组中上调基因168个，下调基因44个。

**结论:**

利用全基因组芯片筛查出212个可能与非小细胞肺癌多药耐药相关的基因，用于指导临床个体化治疗。

## Abstract (en)

**Background and objective:**

The aim of this study is to screen for multi-drug resistance-related genes of human non-small cell lung cancer (NSCLC), and provide the evidences for drug-sensitive predicting genes of different NSCLC patients treated with chemotherapeutic drugs.

**Methods:**

Sensitivity and inhibition ratio of five antitumor drugs (NVB, GEM, TAL, DOC, CDDP) on 75 fresh NSCLC samples from different individuals were studied by means of culturing primary tumor cells and MTT assay. After the five chemotherapeutic drugs were used, multi-drug resistance-related genes of NSCLC with cDNA microarry on the samples which were all high sensitive and those resistant were screened.

**Results:**

cDNA microarray analysis screened out 212 genes, 168 of which were up-regulated while the other 44 were down-regulated in the group of highly sensitive compared with the group of resistance.

**Conclusion:**

The multi-drug resistance of NSCLC may be correlative with the 212 genes screened by cDNA microarray; the detailed mechanisms of the genes still need to be detected in the future.

肺癌是目前世界上死亡率最高的恶性肿瘤之一，与20世纪初相比，现在肺癌已经成为威胁人类健康和生命的最大敌人之一，其5年生存率仅为15%左右^[1]^。70%-80%的肺癌患者在确诊时已属晚期，化疗是最主要的治疗手段之一。肿瘤细胞对抗癌药物产生耐药性是导致化疗失败的主要原因。肺癌耐药与多种基因的表达异常相关，全面揭示化疗耐药的分子学变化特征，并筛选出与药物敏感性密切相关的基因群即化疗药敏预报基因，对于准确评估患者的化疗敏感性，进而指导临床治疗具有重要意义。

迅速发展的基因芯片技术能从群体水平对非小细胞肺癌（non-small cell lung cancer, NSCLC）的耐药相关基因进行研究。本研究收集临床手术切除的NSCLC组织进行原代培养和药物敏感性测定，筛选出对5种化疗药物（诺维本、吉西他滨、多西他赛、紫杉醇及顺铂）耐药或敏感的组织，以cDNA芯片分析基因表达谱的变化，筛查耐药相关的基因，为寻找化疗药敏预报基因提供实验依据。

## 1 材料和方法

### 1.1 材料

黑龙江省肿瘤医院手术室2007年11月-2009年8月的病理分型确定且术前、术中未行放、化疗的NSCLC组织75例，于取样1 h内进行试验。

### 1.2 主要试剂和仪器

胎牛血清（杭州四季青生物工程材料有限公司），MTT粉（AMRESCO公司），RPMI-1640培养液（GIBCO公司），酶标仪（美国BioTeK公司）。人类全基因组表达谱寡聚核苷酸芯片Human Genome U133 2.0 plus Affymetrix购自美国公司，该芯片含47 400个转录本，共代表38 500个基因。基因芯片主要试剂包括：Eukaryotic Poly-A RNA Control Kit、One-Cycle cDNA Synthesis Kit、GeneChip IVT Labeling Kit、GeneChip Sample Cleanup Module均为Affymet rix公司产品。

### 1.3 人NSCLC细胞的原代培养

超净台内去除肿瘤组织血管、结缔组织和坏死组织，PBS将肿瘤组织清洗至透明后，移至含有0.25%浓度胰蛋白酶液的平皿中，边消化边剪，尽量将肿瘤组织剪碎至糊状，再用玻璃注射器芯经200目的不锈钢网筛轻轻研磨，边研磨边滴加RPMI-1640培养液边过滤。将得到的细胞液经肿瘤细胞分离液离心（1 000 rpm, 5 min），小心地吸取中层的絮状物质，即肿瘤细胞，将收集得到的肿瘤细胞用1640培养液混匀制成肿瘤细胞悬液，用计数板细胞计数，调整活细胞数1×10^5^/mL。取部分细胞加入Trizol吹打均匀，存放于-80 ℃冰箱中。

### 1.4 MTT法检测5种化疗药对原代人NSCLC细胞的细胞毒作用

将各种肺癌细胞系悬液以1×10^5^/mL接种于96孔微量培养板中，190 μL/孔，将96孔板放入37 ℃、5%CO_2_、饱和湿度的二氧化碳培养箱中培养，待细胞贴壁后，加入7种浓度的抗癌药各10 μL（根据分别的临床血浆高峰浓度配置成7种浓度：分别为10×ppc、5×ppc、2.5×ppc、1.25×ppc、0.625×ppc、0.312 5×ppc、0.156 25×ppc）（①NVB；②GEM；③DOC；④TAL；⑤CDDP），设4个复孔；同时设细胞对照孔，只加细胞培养液。将加药后的96孔板放入37 ℃、5%CO_2_、饱和湿度的二氧化碳培养箱中继续培养72 h。培养结束前4 h，每孔加入20 μL MTT（5 mg/mL, PBS），继续培养4 h，取出培养板，1 500 rpm离心5 min，吸弃上清，每孔加入200 μL DMSO，振荡混匀，使甲臜（Formazan）颗粒完全溶解后，以560 nm波长测定光密度（optics density, OD）值，计算抑制率：

抑制率（%）=（对照孔OD值-实验孔OD值）/（对照孔OD值）×100 %。

根据不同浓度梯度的抑制率拟合曲线，选择剂量反应关系良好的标本，以血药峰浓度下细胞抑制率≥50%为高度敏感、30%-50%为中度敏感、≤30%为耐药作为区分标准，挑选对5种化疗药均高度敏感或耐药的样本进行基因芯片试验，并将其分为高度敏感组和耐药组。

### 1.5 标本总RNA提取

将1 mL Trizol分别加入收集好的高度敏感组和耐药组细胞中，充分裂解后加入250 μL氯仿，离心后取上清液至一新的离心管中，加入500 μL异丙醇，室温沉淀1 h，15 000 g/min离心10 min，此时可见少许白色RNA沉淀紧贴于管壁。弃上清，以1 mL 75 %乙醇洗涤，7 500 g/min离心5 min，将RNA溶解于20 μL DEPC水中，测定浓度和纯度后-80 ℃保存。

### 1.6 基因芯片检测基因表达谱

使用Eukaryotic Poly-A RNA Controls Kit制备合成用Poly-A RNA Controls，制备T7-Oligo (dT)Primer/Poly-A Control Mix。将1 μg RNA样品与T7-Oligo(dT)Primer/Poly-A Control Mix混合，首先使用One-Cycle cDNA Synthesis Kit逆转录合成cDNA，然后进行二链DNA的合成和纯化。体外转录合成生物素标记cRNA。取适量cRNA制备杂交液并与芯片进行杂交过夜。杂交完成后，移除探针阵列中的杂交混合物，利用Fluidics Station 450进行芯片清洗，最后利用Affymetrix GeneChip^®^ Scanner 3000对芯片进行扫描。扫描图像采用Affymetrix GeneChip^®^ Operating Software Version 1.0软件进行数字化处理和分析。

## 2 结果

### 2.1 NSCLC组织标本的收集及药敏试验结果

通过MTT方法挑选对5种化疗药均高度敏感或耐药的样本，并将其分为高度敏感组和耐药组。共选出11例样本，其中高度敏感组6例，耐药组5例（表 1）。

**1:** 化疗药对11例非小细胞肺癌标本细胞的抑制率（%） Inhibition rates of drugs on non-small cell lung cancer cells in 11 specimens (%)

No.		Inhibition rates				
		NVB	GEM	DOC	TAL	CDDP
Resistant	1	21.5	16.9	18.2	30.1	22.0
	2	10.5	14.9	28.6	25.0	15.4
	3	15.0	26.1	19.0	22.2	23.1
	4	22.0	18.5	5.9	35.6	18.0
	5	18.2	10.8	5.3	30.6	15.3
Sensitive	6	50.7	50.2	54.2	63.6	52.2
	7	50.3	53.2	55.1	59.2	55.7
	8	50.1	70.2	63.4	52.5	51.7
	9	50.3	42.4	64.0	58.0	52.4
	10	59.4	59.6	50.4	55.9	64.6
	11	51.3	62.6	51.0	52.4	50.8

### 2.2 基因芯片对耐药和高度敏感NSCLC组织细胞表达差异基因筛查结果

扫描图像采用Affymetrix GeneChip^®^ Operating Software Version 1.0软件进行数字化处理和分析。差异探针筛选标准：|Score(d)|≥2，同时差异倍数即Fold Change控制在2倍以上，样本数≥3。经过软件进行数字化处理和分析后发现，耐药组与高度敏感组差异表达基因一共有212个，占总表达基因数的5.51‰，其中敏感组上调168个，下调44个（表 2）。

**2:** 非小细胞肺癌组织抗癌药物敏感性相关基因的表达状态 Numbers of differentially expressed genes out of 38 500 candidate genes and ratios of expression intensity in non-small cell lung cancer

Gene expression	No. of genes	Ratio of expression intensity (‰)		
		2-3	3-5	> 5
Upregulated	168 (4.36‰)	39 (1.01‰)	110 (2.86‰)	19 (0.49‰)
Downregulated	44 (1.14‰)	30 (0.78‰)	10 (0.26‰)	4 (0.10‰)
Total	212 (5.51‰)	69 (1.79‰)	120 (3.12‰)	23 (0.59‰)

GO分析结果显示，NSCLC组织抗癌药物敏感性相关差异表达的基因包括：具有结合功能蛋白的基因、酶活性相关基因、离子结合相关基因、离子通道及转运蛋白相关基因、氧化还原活性相关基因、细胞骨架蛋白相关基因、应激反应相关基因、信号转导通路相关基因、细胞生长及维持相关基因、细胞周期及调节相关基因、细胞运动及细胞间粘附相关基因、细胞凋亡及调节相关基因等。其中按分子功能分类的结果如（表 3）。

**3:** 非小细胞肺癌细胞株抗癌药物敏感性相关基因差异表达的分类 Category of the differentially expressed genes related to anticancer drug-sensitivity in non-small cell lung cancer

Serial No.	Up-regulated genes			Down-regulated genes	
	Category	No.		Category	No.
1	Binding	72		Binding	33
2	Catalytic activity	42		Catalytic activity	19
3	Moleculartransducer activity	14		Transporter activity	4
4	Signal transducer activity	14		Molecular transducer activity	3
5	Transporter activity	11		Structural molecule activity	3
6	Transcription regulator activity	7		Signal transducer activity	3
7	Enzyme regulator activity	5		Transcription regulator activity	3
8	Electron carrier activity	1		Enzyme regulator activity	1
9	Structural molecule activity	1		Translation regulator activity	1
10	Translation regulator activity	1		----	----

## 3 讨论

化疗是治疗NSCLC的主要方法之一。NSCLC手术切除后应用化疗，可以杀伤残留癌细胞，对防止肿瘤复发和转移具有积极意义。对于失去手术机会的NSCLC患者，化疗可以在一定程度上延缓肿瘤的生长，甚至有可能使肿瘤缩小、为患者争取二期手术的机会。然而，化疗药物普遍存在的多药耐药现象限制了其获得更好的疗效和更广泛的临床应用^[2]^。为了更好地研究NSCLC多药耐药的发生机制，本研究通过基因芯片技术，利用Affymetrix GeneChip^®^ Scanner 3000对芯片进行扫描，筛选可能与NSCLC多药耐药相关的基因，耐药组与高度敏感组差异表达基因一共有212个，其中高度敏感组上调168个，下调44个。这212个差异表达基因中，曾被文献报道过的与耐药相关的基因并不多。

信号转导通路参与了细胞的生长、增殖及凋亡的调控，在生物体内发挥着重要的生理功能。在NSCLC多药耐药的发生过程中涉及到较多的信号转导通路的改变，包括JAK-STAT信号转导通路、TGF-β信号转导通路、p53信号通路等。其中，TGF-β信号通路可以调节细胞的增殖、分化、凋亡、迁移等多种生物过程^[3, 4]^。

SMAD1蛋白是TGF-β信号通路主要的细胞内信号传导分子，已有研究报道在肿瘤中存在SMAD1的异常表达，且SMAD1参与TGF-β信号通路的调节。Liu等^[5]^的研究发现在脑胶质瘤患者中，*SMAD1*基因表达的下调与患者生存期的降低显著相关。本研究耐药组中*SMAD1*基因的表达均下调，预示*SMAD1*基因可能与肿瘤耐药性相关，可通过上调*SMAD1*基因的表达来增强NSCLC化疗的敏感性，提高患者的生存期。

Cathepsin是存在于细胞溶酶体内的蛋白酶类，有B、H、L、D等亚类。Cathepsin可通过多种路径引起细胞的凋亡^[6]^。Cathepsin B和Cathepsin L与恶性肿瘤的转移和癌症的进展密切相关^[7-9]^。Reisenauer^[10]^认为，Cathepsin B活性的增加可抑制细胞凋亡，并伴有TGF-β信号通路中*SMAD1*基因的表达下调。本试验结果中敏感组*SMAD1*基因表达上调，同时Cathepsin B基因表达下调，而耐药组Cathepsin B基因表达上调。所以通过抑制Cathepsin B基因的表达可能会降低肿瘤的耐药性，提高化疗的疗效。

AZGP1（alpha-2-glycoprotein 1, zinc-binding）即锌α2-糖蛋白（ZAG）中是40 kDa的单链多肽^[11]^。在Albertus^[12]^的研究中发现，40%肺腺癌患者的血清中检测到AZGP1自身抗体，而自身抗体的产生与5年生存率的提高显著相关。Henshall^[13]^的研究表明，AZGP1的低表达与前列腺癌的临床复发、骨转移和生存显著相关，在17例短期内即出现复发的前列腺癌的患者中，其中13例存在*AZGP1*基因表达减弱或缺失的现象。上述结论表明恶性肿瘤患者中AZGP1的降低提示预后不良，本研究结果显示在敏感组细胞中的AZGP1呈高表达状态，差异倍数达到31.2，而在耐药组细胞中呈低表达状态，提示AZGP1的低表达可能与耐药机制相关，从而引起预后不良，但尚需进一步实验研究证实。

NSCLC化疗耐药是一个多基因、多环节、多途径参与的过程，可能涉及影响不同生化途径的多种遗传因子表达的改变。本研究利用全基因组芯片高灵敏度、高通量的特点，初步筛选出一系列可能与NSCLC多药耐药相关基因。并结合生物信息学找出可能与肿瘤药物敏感性和耐药性相关的基因，下一步将对差异基因做进一步的验证，寻找出与肿瘤化疗敏感性和耐药性相关的分子标志物，构建NSCLC多药耐药基因相关的cDNA文库。
